# Boils and Whitlows

**Published:** 1879-09

**Authors:** 


### BOILS AND WHITLOWS

are relieved or dissipated in their earlier stages by using the tincture of camphor. Dip a finger in the camphor and rub it over the boil ; do this eight or ten times, and repeat every four hours during daylight.

For whitlow, dip the finger into the camphor and let it remain ten minutes ; this often gives immediate relief. Repeat every three hours during the day until cured, eating nothing, meanwhile, but coarse bread and butter and fruits. Prepare the camphor thus : Put an ounce or more in a vial, fill with alcohol, shake it well ; some of the camphor should always be seen at the bottom ; this ensures a saturated tincture, which is the strongest.

